# Robot-Assisted 2D Fluoroscopic Needle Placement—A Phantom Study

**DOI:** 10.3390/diagnostics14161723

**Published:** 2024-08-08

**Authors:** Yannick Scharll, Nenad Radojicic, Gregor Laimer, Peter Schullian, Reto Bale

**Affiliations:** Interventional Oncology-Stereotaxy & Robotics (SIP), Department of Radiology, Medical University Innsbruck, Anichstr. 35, 6020 Innsbruck, Austria

**Keywords:** imaging, interventional, robotics, robotic surgical procedures, punctures, fluoroscopy

## Abstract

Rationale and Objectives: To evaluate the targeting accuracy of a novel robot-assisted guidance technique relying on one pair of 2D C-arm images. Material and Methods: In total, 160 punctures were carried out semi-automatically by using a novel robotic device. The needle’s paths were planned based on one pair of 2D fluoroscopic images from different angles. Conically shaped aluminum tips inside a gelatin-filled plexiglass phantom served as targets. The accuracy of the needle placement was assessed by taking control CTs and measuring the Euclidean distance (ED) and normal distance (ND) between the needle and the target point. In addition, the procedural time per needle placement was evaluated. Results: The accomplished mean NDs at the target for the 45°, 60°, 75° and 90° angles were 1.86 mm (SD ± 0.19), 2.68 mm (SD ± 0.18), 2.19 mm (SD ± 0.18) and 1.86 mm (SD ± 0.18), respectively. The corresponding mean EDs were 2.32 mm (SD ± 0.16), 2.68 mm (SD ± 0.18), 2.65 mm (SD ± 0.16) and 2.44 mm (SD ± 0.15). The mean duration of the total procedure, including image acquisition, trajectory planning and placement of four needles sequentially, was 12.7 min. Conclusions: Robotic guidance based on two 2D fluoroscopy images allows for the precise placement of needle-like instruments at the first attempt without the need for using an invasive dynamic reference frame. This novel approach seems to be a valuable tool for the precise targeting of various anatomical structures that can be identified in fluoroscopic images.

## 1. Introduction

Interventional procedures can be guided by a variety of imaging modalities. Ultrasound is generally available and radiation-free, and it provides real-time image guidance. However, many lesions are not accessible by ultrasound guidance due to having a deep location or shadowing artefacts caused by air, bone or bowel. Alternatively, computed tomography (CT), cone-beam CT and magnetic resonance tomography (MRI) may be used for image guidance. CT-guided interventions including biopsy, sympathicolysis, nerve root infiltration and facet joint injection are increasingly performed by interventional radiologists [1]. However, only a few centers have CT, MRI or cone-beam CT in their operating theatres available. Therefore, most orthopedic surgeons still rely on standard fluoroscopic imaging.

A variety of needle guidance and placement assistance systems have been developed for percutaneous image-guided procedures. They include optical [2] and electromagnetic navigation systems [3], laser overlay systems [4], ultrasound (US)-guided systems [5] and robotic systems [6]. Great efforts have been put into the development of robotic systems. Robots are programmable, multifunctional specialized devices that may be used in a diversity of fields [7]. Despite their use in disciplines such as neurosurgery, orthopedics, and urology, medical robots are not the standard of care, and market penetration has been very limited. The limited use of robotic systems in medicine can be attributed to the many challenges associated with developing a robotic system, particularly ensuring patient safety. Accuracy is of paramount importance during robotic procedures, as it directly impacts patient outcomes and procedural success. By using precise robotic movements, instruments can be positioned and maneuvered precisely, allowing them to minimize the risk of damage to surrounding tissues and vital structures. Previous studies have demonstrated the high accuracy of robot-assisted CT-guided punctures [2,8], and Mbalisike et al. [9] demonstrated that robotic-guided approaches improve accuracy in localizing tumors compared to manual methods.

The novel and compact-designed robotic guidance system presented in this study was previously combined with an optical camera and yielded satisfactory accuracy results for CT-guided needle placements [10]. In a second workflow, the robotic system is compatible with a C-arm, so needle punctures can be performed based on two-dimensional (2D) X-ray images. The purpose of this initial phantom study was to determine the accuracy and practicability of the fluoroscopic workflow before introduction into clinical practice.

## 2. Materials and Methods

### 2.1. Phantom

**Figure 1:**
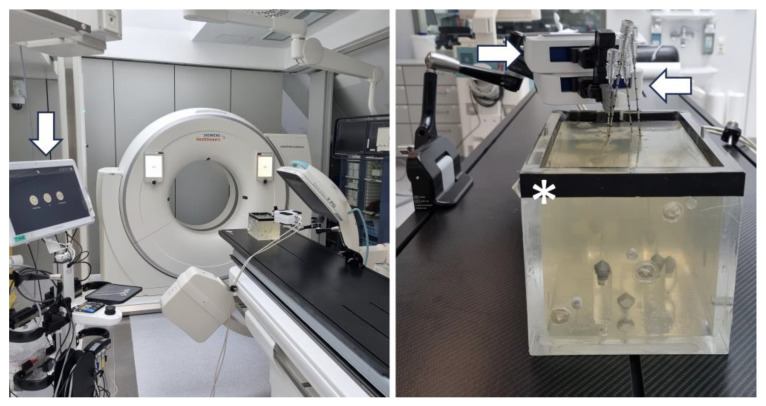
Experimental setup showing the phantom (*) centered below the C-arm. The robotic device consists of two parts: the lower positioning part (POS, ←) and the upper angulation part (ANG, →). The planning monitor (↓) can be operated via touchscreen.

Our custom-designed phantom consists of a box made of plexiglass (220 × 150 × 175 mm, Figure 1). The tips of eight radio-opaque aluminum cones serve as target points. They are glued on top of plexiglass cylinders, which extend from the bottom of the cube with varying heights. In order to stabilize the needles during the time from needle placement to CT evaluation, the phantom is filled with gelatin and cooled to 4° Celsius. Gelatin-filled phantoms can be used to perform reproducible accuracy studies on image-guided needle placements [11]. Various other guidance systems have already been evaluated using a similar phantom [2,3,8,12,13,14].

### 2.2. Robotic Navigation System

The Micromate (iSYS Medizintechnik GmbH, Kitzbühel, Austria) surgical robotic system consists of a four-degrees-of-freedom robot needle positioning unit mounted on a passive holding arm with seven degrees of freedom. It comprises two modules: the lower part (POS) and the upper part (ANG). In POS, the end effector can be positioned longitudinally and transversally, restricted to a rectangular field of approximately 40 mm by 40 mm (X × Y). In ANG, the end effector can be angulated around 2 axes, specifically around a frontal horizontal axis and a sagittal horizontal axis, thereby allowing angular deflections ranging from −30 to +30 degrees. In order to put the manipulator in the final target aiming position, 2D or three-dimensional (3D) intraoperative images may be used. The registration process in this study relies on a pair of 2D fluoroscopy C-arm (Ziehm Vision RFD 3D, Nürnberg, Germany) images, including one anterior–posterior (AP) and one lateral–lateral (LL) view. Two different angles can be freely chosen for the C-arm in order to be able to obtain sufficient information for 3D surgical planning. For best results, angle selection should be made based on the target’s location. To identify and register the robotic device in relation to the phantom, 5 spherical radiopaque markers are integrated in the radiolucent end effector serving as reference points for automatic registration. To achieve robot-to-image-space registration, the end effector must be in the field of view (FOV) of the fluoroscopy. The needle holder is attached to the end effector and allows us to guide the puncture needle to the target.

### 2.3. Experimental Workflow

**Figure 2:**
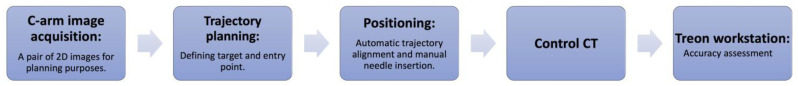
Operational flow.

**Figure 3:**
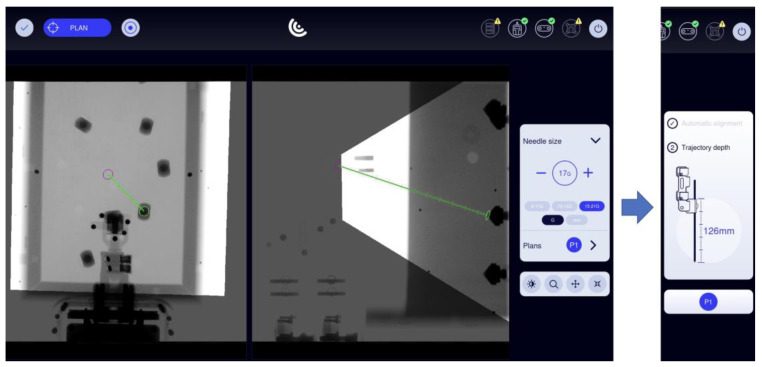
Screenshot from the Micromate workstation (Micromate Naviplus+) showing the AP image on the left and the LL image (90°) on the right. After selecting the needle size, the target point (green circle) and the entry point (purple circle) can be selected by “drag and drop” via touchscreen. Following confirmation of the path, the robot can perform automatic alignment and display the needle’s length.

The robotic device was grossly positioned above the phantom using the passive holding arm. The angle of the LL image was changed in four series of 40 punctures each (45°, 60°, 75° and 90°) relative to the strictly vertically oriented AP image, which was regarded as the 0° starting point (Figure 2). Automatic registration was achieved by taking two fluoroscopic images from AP and LL views. By using these 2D images, the virtual needle paths were defined. Planning was carried out on the workstation’s touch screen by using the Micromate planning software (Micromate Naviplus+, vers. 1.2.3, Figure 3). The target and entry points were selected on the images. For this study, the aluminum tips inside the phantom served as target points. A random entry point on the gel surface was chosen to allow for a variety of different double oblique needle paths. Positional information about the selected trajectory was transformed into robot coordinates. The robot unit was then activated and moved automatically to the correct location. The navigation system’s planning software determined the insertion depth, and the 17-gauge needle (with a length of 200 mm) was manually inserted into the preplanned depth.

### 2.4. Evaluation

**Figure 4:**
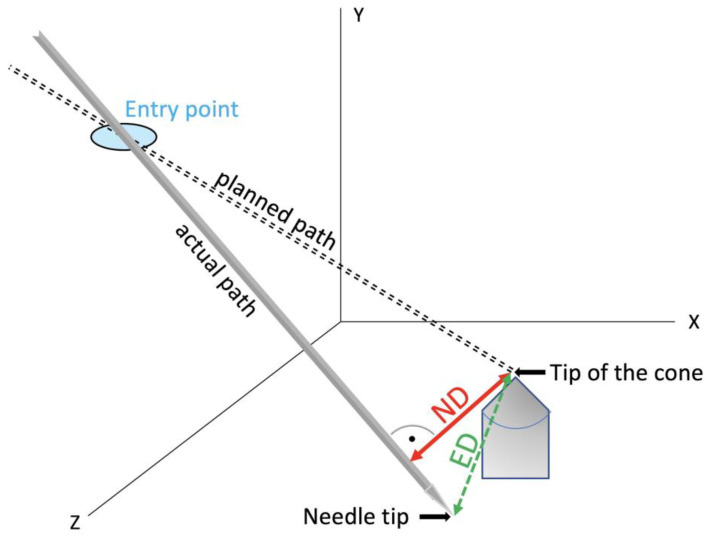
In a multidimensional space, the ND describes the shortest possible distance between a point and a straight line. On the other hand, the ED indicates the distance between two points, meaning the actual position of the needle tip and the target point.

Post-procedural CT scans in 1 mm slice thickness were obtained to evaluate accuracy after needle placement. The volumetric data sets were transferred to the Treon Stealth Station (Medtronic, Inc., Dublin, Ireland). The software “Mach Cranial, vers. 5” provided by the device was used for accuracy assessment. In this study, the coordinates of the needle tip, the cone tip and each probe’s entry point were determined. Using basic analytical geometry formulas [15], normal distances (NDs) between the needle axis and the target, and Euclidean distances (ED) between the target and positioned needle tips (Figure 4), were calculated. The mean errors, standard deviation, maximal values and minimal values were determined. In addition, the average duration of the total procedure for one needle placement was evaluated.

SPSS Version 24 (SPSS Inc., Chicago, IL, USA) was used to conduct the statistical analysis. The mean errors and the standard deviation were calculated. The difference between continuous variables was evaluated using a one-way ANOVA with Bonferroni’s post hoc test. A *p*-value of <0.05 was considered statistically significant.

## 3. Results

**Figure 5:**
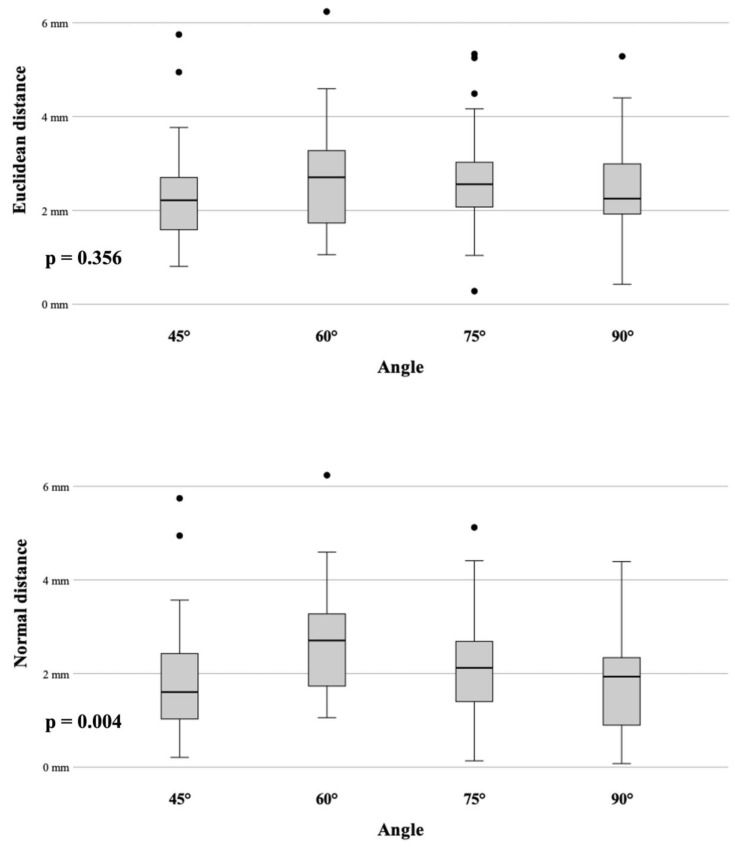
Box plots displaying the targeting accuracy (ED and ND) for each angle.

In total, 160 punctures were carried out semi-automatically by using this novel robotic device. We divided the measurements into four groups based on the angle settings for the C-arm in each group. With a mean root-mean-square (RMS) registration error of 0.15 mm, the accomplished mean NDs at the target for the 45°, 60°, 75° and 90° are summarized in Table 1 and Figure 5.

### 3.1. Angle Accuracy Comparison

The Bonferroni post hoc test showed no significant difference in accuracy comparing the ED results. On the other hand, a significant difference in accuracy for the ND was observed between 60°/45° (*p* = 0.01) and between 60°/90° (*p* = 0.01).

### 3.2. Accuracy and Target Depth

The mean depth of the target (distance between the needle entry point and the target) for all 160 punctures was 74.88 ± 11.34 mm (range 55–107 mm). The punctures were categorized into two groups, the first one with a target depth of ≤75 mm and the second group with a target depth greater than 75 mm. According to the *t*-test, the target depth did not affect the accuracy by comparing the two groups (*p* = 0.147 for ND and 0.133 for ED). Furthermore, by examining each group separately, the target depth had no significant impact on the accuracy of the ND and ED:

ND: 45° (*p* = 0.525), 60° (*p* = 0.732), 75° (*p* = 0.175), 90° (*p* = 0.059)

ED: 45° (*p* = 0.813), 60° (*p* = 0.732), 75° (*p* = 0.241), 90° (*p* = 0.077)

### 3.3. Procedural Time

The mean duration of the total procedure, including the image acquisition, trajectory planning and placement of four needles sequentially, was 12.7 min. It comprises a mean image acquisition time of 6.8 min, a mean trajectory planning time of 3.1 min and a mean positioning time of 2.8 min, including the alignment of the robot and needle insertion. Table 2 details the time measurements for each angle.

## 4. Discussion

The popularity of minimally invasive percutaneous surgical procedures is on the rise due to their less invasive nature compared to traditional open techniques. Various medical specialties are utilizing computer-based imaging systems for non-invasive or minimally invasive procedures. In imaging-guided minimal invasive procedures, surgeons depend on indirect views from imaging modalities as they cannot directly visualize organ tissues.

### 4.1. Imaging Modalities

Through technological advancements, existing imaging modalities have improved, as well as new ones emerging, each offering a different set of features. CT and MRI are commonly used imaging modalities for acquiring pre-interventional images as they have high spatial resolution and versatility. Ultrasound imaging and incremental CT imaging enable real-time intraoperative imaging due to their fast acquisition of images, commonly used for targets prone to movement. Moreover, fluoroscopy units, commonly referred to as C-arms, play an important role in today’s modern operating rooms and provide real-time intraoperative 2D visualization while ensuring efficiency and precision.

### 4.2. Robotic Systems

Currently, physicians face unique engineering challenges and new knowledge demands due to the combination of image-guided surgery and robots with the complexity of soft tissue registration, operative navigation and surgical use. In recent years, robotically guided interventions have made it easier to place needles or instruments during surgery or interventional procedures [7,16,17,18,19,20]. They support interventions under CT, MRI and US guidance [21]. A major application of widespread CT-guided robotics is the positioning and insertion of needles for biopsies or therapeutic procedures, and numerous studies have demonstrated its effectiveness. Comparatively, robotic interventions guided by MRI have made few advances, since MRI-compatible systems require complex design and development. Nevertheless, studies have shown their capability for puncturing targets, for example, during prostate [22] and breast [23] biopsies or the treatment of renal cancer [24]. For robotic assistance in US imaging, highly sensitive systems are required to allow for direct human–robot interaction. In the past, various systems have been described that include features such as automatic surface reconstruction and automatic probe positioning. As an example, Berger et al. [25] achieved positioning errors from 1.69 ± 0.92 to 1.96 ± 0.86 mm in an abdominal phantom.

### 4.3. Combination of Robotic Device with C-Arm

Only a few studies have investigated the hybrid approach using a C-arm and a robot, despite the popularity of these devices. The capabilities of C-arm imaging range from distorted 2D planar images to cone-beam computed tomography (CBCT). Czerny et al. [26] combined a robotic device using C-arm cone beam CT (3D preoperative images) for needle guidance during spinal interventions. An average deviation of 0.35 mm was recorded between the planned path and the K wire during percutaneous placements.

In a recent study, it was demonstrated that 2D data are sufficient for robot-to-image-space registration. In a porcine model, Kim et al. [27] compared planned pathways with placed pathways to determine pedicle screw accuracy using 2D digital spot fluoroscopy in combination with a robotic device and optical tracking. Pedicle placement was executed with an average offset up to 5.3 ± 2.3 mm.

The present study highlights the performance of a novel robotic guidance system combined with a C-arm only using 2D images. In contrast to the previously mentioned study, no additional sensors (e.g., infrared or laser) are required. To the best of our knowledge, no previous experimental study has reported applying this workflow.

### 4.4. Accuracy

A major purpose of our study was to evaluate the new image-based guidance system’s accuracy. Results were reproducible and accurate within a range of 1–3 mm. The angle setting does not have a significant impact in accuracy when comparing the ED results. The ND results for the 60° group, however, were significantly worse than those for the 45° and 90° groups (*p* = 0.01), even though they remained within an acceptable range. Although the needle insertion depth is calculated by the software of the robot and indicated on the device, overshooting the target might be prevented by the aluminum tip. The conical shape of the target body can deflect the needle tip and consequently lead to an additional lateral deviation, resulting in a false increase in the ND. In clinical practice, overshooting the target can be prevented by performing fluoroscopic imaging close to the target and corrected easily by retracting the needle. In cases of an angular deviation of the probe, manual re-angulation or re-insertion may be required. Thus, the normal distance (ND) is the most crucial factor. This study’s results are difficult to compare with those of existing studies on robotic devices for percutaneous interventional procedures. In fact, most of them use different experimental designs and phantoms, even evaluating different endpoints. A similar phantom and post-procedural workflow for assessing accuracy was applied to eight different navigation systems [2,3,8,10,12,13]. In a previous ex vivo study [10], the Micromate system was combined with optical tracking, and the accuracy was assessed during CT-guided needle placements, resulting in a mean ND for the thinnest CT slice (1 mm) of 1.34 mm (SD ± 0.82). These results are comparable to other robotic systems, e.g., the Maxio robot (Perfint Healthcare, Chennai, India) with a mean ND of 1.3 mm (SD ± 0.8) or the robotic assistance system Innomotion (Innomedic GmbH, Herxheim, Germany) achieving a mean ND of 1.6 mm (SD ± 0.9) [8,12]. Although the studies were limited to a CT workflow using volumetric DICOM formatted images for planning, the average deviations between the planned target and needle tip are comparable to that of this study.

By comparing our results to previous reviews about robotic systems [18,28], the high accuracy of this approach is due to the simplicity of the experimental setup. In fact, the target registration error is limited to technical errors in the computer-assisted position measurement and errors in the definition of the radiopaque markers, delivering a mean RMS error of 0.15 mm. Additional inaccuracies occur when, e.g., additional infrared markers have to be detected in the context of optical tracking. The method requires no additional sensors (infrared, laser, ultrasound, electromagnetic, etc.), no stereotactic frame and no prior calibration.

Identifying the target point is certainly challenging when dealing with two-dimensional data sets. This study used aluminum cone-shaped targets, which simplify planning. A possible clinical application of this technique is to place pedicle screws [27] or treat fractures [29,30], which mainly limits it to the field of neurosurgery and orthopedics. Another application would be the retrograde drilling of osteochondral lesions of the talus [31] or the percutaneous management of metastatic osseous disease [32,33,34]. It is important to keep in mind that image quality varies greatly depending on the target anatomy, and identifying anatomical landmarks can be problematic.

An increase in needle placement accuracy may reduce the likelihood of complications. A needle misplaced in a small bone is more likely to result in the destruction of healthy bone, and healing might be worse [29]. High accuracy may reduce the number of trials for perfect implant placement without adding radiation or extending the procedure. The obvious advantages that the robot systems offer are stability and repeatability. Compared to the conventional freehand techniques, robotic systems are presumably equally accurate for both simple and complex angles and less dependent upon physician comfort and skill. As compared with other navigation tools, an advantage of the guidance system set-up tested in this study is its ability to be integrated into existing workflows. In fact, most hospitals are using C-arms and fluoroscopy as a very cost-effective modality. The investigated system does not rely on costly disposables such as electromagnetic or optically tracked needles. Furthermore, there is no need for extensive pre-procedural registration, which can be time-consuming if fiducial markers and EM fields (or cameras for optical tracking) are to be identified intra-procedurally.

One of the major limitations of the workflow is the need to depict the end effector with its markers on each C-arm shot. Therefore, it depends on the surgical area as to whether the system can be used. It is essential to mount the device above the patient with an approximate prior knowledge of the entrance and target location (trajectory). In fact, the robotic device must be manually prepositioned near the target in a rough alignment with the trajectory before end-effector localization can occur; yet, the robot’s compact design stands out and facilitates the procedure. During the study, it was not always possible to obtain a perfect X-ray image on the first try leading to noticeable differences in acquisition times. The reference-free design does not allow for the real-time tracking of the needles position and orientation despite the given information of the needle depth. It is not possible for the system to compensate for respiratory movements of the target region. It is important to immobilize patients in a stable manner in order to decrease the risk of injury during unpredictable patient movement. However, the needle can be easily detached from the needle holder, and the risk of unintentional injury during the movement of the robot is minimal because two keys have to be pressed and held simultaneously in order to move the robot.

In conclusion, the compact design of the robot and the simplicity of the workflow seem to be attractive. Mapping the end effector by two fluoroscopic images from different angulations was sufficient to register the robot to the target. As a result, the procedural time was relatively short. To the best of our knowledge, this workflow has not been described before. The novel robotic device in combination with 2D fluoroscopy (C-arm) provides stable and accurate needle guidance, even for double-oblique angulated approaches. The system showed satisfactory accuracy for executing the intended planned trajectory and could be useful in a variety of orthopedic procedures. To establish this novel system in the clinical routine, further experiments in cadavers and in the clinical setting will be required.

## Figures and Tables

**Table 1:** ED and ND accuracy results.

Angle	ED	ND
45°	2.32 mm (SD ± 0.16)	1.86 mm (SD ± 0.19)
60°	2.68 mm (SD ± 0.18)	2.68 mm (SD ± 0.18)
75°	2.65 mm (SD ± 0.16)	2.19 mm (SD ± 0.18)
90°	2.44 mm (SD ± 0.15)	1.86 mm (SD ± 0.18)

**Table 2:** Procedural durations in minutes (range).

Angle	Image Acquisition	Trajectory Planning	Positioning	Total
45°	5.2 (2.9–7.5)	3.5 (2.7–5.0)	2.7 (2.3–3)	11.4 (10.1–13.5)
60°	9.2 (4.7–16.6)	3.1 (2.8–3.5)	2.7 (2.4–3.1)	15.1 (10.2–22.6)
75°	6.9 (3.1–16.9)	3.1 (2.2–3.5)	3.0 (2.3–3.2)	13.0 (9.3–22.9)
90°	5.7 (2.5–10.4)	2.7 (1.1–4.0)	2.9 (2.4–3.1)	11.4 (8.0–16.8)

## Data Availability

The data that support the findings of this study are available from the corresponding author, BR, upon reasonable request.
